# Contemporary patellofemoral arthroplasty is associated with lower revision rates compared with the previous decade: A meta‐analysis using revisions per 100 component‐years

**DOI:** 10.1002/ksa.70381

**Published:** 2026-03-10

**Authors:** Clemens Clar, Maximilian Budin, Laura Rasic, Andreas Leithner, Julia von der Linden, Patrick Sadoghi

**Affiliations:** ^1^ Department of Orthopaedics and Trauma Medical University of Graz Graz Austria

**Keywords:** patellofemoral arthroplasty, patellofemoral joint, survival curve, survival rate, revision rate

## Abstract

**Purpose:**

The aim of this study was to assess global data on patellofemoral arthroplasty (PFA) survival a decade after a prior investigation by Reihs et al. using the same endpoints and data sources, aiming to quantify potential changes in implant survival over this period. The hypothesis was that the pooled revision rate of contemporary PFA, quantified as revisions per 100 component‐years (CY), would be lower than prior decade estimates derived from identical methodology and registry sources.

**Methods:**

A systematic search in Embase, MEDLINE and CENTRAL through October 2025 using a predefined search strategy was conducted using Ovid. Two reviewers screened and extracted data. The primary effect measure was revisions per 100 CY. Pooled estimates were obtained with fixed‐ and DerSimonian–Laird random‐effects. Risk of bias was assessed with ROBINS‐I. National arthroplasty registries meeting EAR/EUPHORIC Type A.1.1.1.1. criteria were analyzed.

**Results:**

Fifteen primary studies were included, with most series using contemporary onlay designs. Per‐study rates ranged from 0.355 to 3.400/100 CY. The pooled revision rate was 1.59/100 CY (95% confidence interval [CI] = 1.24–2.04) with random effects, corresponding to a 10‐year revision rate of 15.9%, representing an absolute improvement of 6.3% in survivorship compared to the benchmark analysis by Reihs et al. heterogeneity was moderate (*I* ^2^ = 52.7%). ROBINS‐I indicated predominantly critical risk of bias. Registry synthesis (NJR, NZJR, AOANJRR, 117.524 CY) yielded 1.85/100 CY (95% CI = 1.775–1.931), corresponding to a 10‐year revision rate of 18.5% (95% CI = 17.75–19.3) and an improvement of 0.4%–8.5% in the last decade if compared to Reihs et al.

**Conclusions:**

This meta‐analysis demonstrates that modern PFA has a more favourable survivorship profile over the past decade, particularly with onlay designs. Proper patient selection and registry‐based surveillance remain key to further optimization.

**Level of Evidence:**

Level IV.

**Abbreviations**
AOANJRR: Australian Orthopaedic Association National Joint Replacement Registry
CI: confidence interval
CY: component‐years
EFORT: European Federation of National Associations of Orthopaedics and Traumatology
NJR: National Joint Registry
NZJR: New Zealand Joint Registry
PFA: patellofemoral arthroplasty
PFJ: patellofemoral joint
TKA: total knee arthroplasty
UKA: unicondylar knee arthroplasty

## INTRODUCTION

Contemporary studies suggest that 11%–24% of patients over the age of 55 presenting with knee pain suffer exclusively from isolated osteoarthritis of the patellofemoral joint (PFJ), for which treatment options include total knee arthroplasty (TKA) as well as patellofemoral arthroplasty (PFA) [7, 11, 15, 37]. The advantages of PFA entail preservation of natural knee kinematics, shorter hospital stays, reduced invasiveness and faster postoperative recovery [21, 40]. Since its introduction in 1955, PFA has evolved significantly. Early studies indicate revision rates of up to 35% prompted critical reassessment [5], whereas subsequent improvements in implant design and patient selection have been associated with reported survivorship of 92% at 5 years, 83% at 10 years, 75% at 15 years and 67% at 20 years [20, 45].

National arthroplasty registries provide population‐level outcome data; however, their interpretation remains limited by relatively low procedural volumes for PFA [8]. To date, no contemporary analysis has reassessed global PFA revision rates using the same endpoint definition and registry sources as the benchmark study by Reihs et al., thereby enabling direct comparison across decades. Such a methodologically consistent reassessment provides surgeons and healthcare systems with an updated benchmark for contemporary implant durability and supports evidence‐based decision‐making when considering PFA. Accordingly, a systematic review approach was employed, integrating registry data with recent clinical studies for comprehensive analysis [1, 4, 25, 30].

The aim of this study was to assess global data on PFA survival a decade after a prior investigation by Reihs et al. applying identical endpoints and registry sources to enable temporal comparison. The hypothesis was that the pooled revision rate for contemporary PFA, quantified as revisions per 100 component‐years (CY), would be lower than prior decade estimates derived from identical methodology and registry sources.

## MATERIALS AND METHODS

The online databases Embase, Medline and Central were systematically screened using Ovid. To minimize bias, study headings, medical subheadings and keywords were refined using Boolean operators. Subsequently, the search terms were adapted according to the specific characteristics of each database. The applied search terms were: ((knee joint OR patellofemoral joint OR osteoarthritis, knee OR patella OR femur) AND (arthroplasty, replacement, knee OR prosthesis failure OR prosthesis‐related infections) AND (patellofemoral OR femoropatellar)) NOT (‘TKA’ OR ‘total knee’ OR ‘unicondylar’).

This meta‐analysis was preregistered on PROSPERO and conducted in compliance with PRISMA guidelines [29].

The databases were screened and assessed for eligibility by two independent individuals (L.R. and C.C.). Potential overlap of patient cohorts was evaluated by comparing study characteristics, including institution, recruitment period, sample size and author groups. When overlapping cohorts were identified, solely the most recent study or the dataset with the longest follow‐up period was incorporated in the meta‐analysis to avoid double‐counting of patients. To be included in the study, publications had to meet the following criteria: a mean follow‐up period of at least 24 months, a clearly stated endpoint of revision for any reason, defined as the explantation or exchange of at least one prosthetic component, and a specified implant type, with unicondylar knee arthroplasty (UKA) and TKA being excluded. Studies were excluded if they were case reports or re‐revision studies [40]. Publications from January 2015 onward were eligible for inclusion, and the final database search was conducted in October 2025.

The following parameters were analyzed: title, authors, published journal, number of primary surgeries, type of prosthesis, patient follow‐up duration (in months), number of patients lost to follow‐up and deceased patients, number of revisions for any reason and revision rate.

Data from national arthroplasty registries were accessed through the official EFORT (European Federation of National Associations of Orthopedics and Traumatology) website for European arthroplasty registries and through official national websites for the rest of the world [14]. A total of 24 national registries were identified, of which only 11 provided data on knee arthroplasty. These databases were subsequently screened for available data on PFA, with inclusion criteria requiring information on the number and model of PFAs used, the number or rate of revisions and the follow‐up period. Ultimately, only three registries, the National Joint Registry (NJR) of the United Kingdom, the New Zealand Joint Registry (NZJR) and the Australian Orthopaedic Association National Joint Replacement Registry (AOANJRR), met all the requirements and were included in the study. These registries were classified as Type A.1.1.1.1., corresponding to the highest level of registry reporting according to the EAR/EUPHORIC classification criteria [23, 33, 40].

### Parameters compared in the study

In this study, no outcome analysis of a single implant was performed. Instead, inventor studies versus independent studies and onlay versus inlay designs were compared, and a comparative analysis of the total survival rates was conducted. Furthermore, mid‐term (≤7 years) versus long‐term studies (>7 years) were analyzed and compared. Publications that indicated the centre of origin of an implant or named its developers as authors or co‐authors were categorized as ‘developer studies’ [40].

### Assessment of revision for any reason

The primary outcome was defined as ‘revision for any reason’. This parameter serves as an objective measure, encompassing a wide range of complications following PFA that ultimately necessitate reoperation of the prosthesis [38]. It indicates the risk of arthroplasty revision over specific time periods, thereby enhancing the comparability of the included studies [33, 40].

### Revisions per 100 observed CY

The data from the three annual reports of the prosthesis registers and the included studies were pooled, and the revision rate was calculated as ‘revisions per 100 observed CY’. This is a widely used classification of this outcome in the field of orthopaedics [33, 34, 45]. The revision rate per 100 CY was calculated as the number of revisions divided by the total observed CY, multiplied by 100. Mathematically, a value of 1 revision per 100 CY correlates with a revision rate of 10% within 10 years [16, 33, 40]. In this study, the value of revisions per 100 CY is used as an indicator to compare outcomes between the individual studies and groups. This enables a standardized statement about the outcomes, despite the varying follow‐up periods of the individual studies. By calculating the revision rate in CY, it is possible to establish a constant hazard rate over the entire investigation period and present the results in a statistically significant manner. The calculation of revision rates using the Kaplan–Meier method was not feasible, as most included studies did not provide extractable time‐to‐event data. Only Akhbari et al. [2] reported both a Kaplan–Meier curve and life‐table information, while a few others mentioned Kaplan‐Meier analyses but lacked sufficiently standardized or complete reporting to allow consistent reconstruction of time‐to‐event data required for pooled Kaplan–Meier analysis [8, 40].

### Assessment of quality

The ROBINS‐I tool was used for the assessment of the quality of the included studies. Among the 15 included studies, the following seven domains were categorized as ‘low risk’, ‘moderate risk’, ‘serious risk’ or ‘critical’: bias due to confounding, bias in the selection of participants into the study, bias in the classification of the intervention, bias due to deviations from intended interventions, bias due to missing outcome data, bias in the measurement of the outcome and bias in the selection of the reported result [44].

### Statistical analysis

IBM SPSS Statistics 21 (SPSS Inc.) was used for statistical analyses. The investigated parameters were evaluated either as absolute numbers or as medians. The included parameters were analyzed descriptively, as no *p* values could be calculated because the registry data and clinical studies report real‐world outcomes rather than probabilistic estimates. Differences between groups were therefore interpreted in a descriptive manner, acknowledging that revision rates after PFA are influenced by multiple factors independent of the implant used. Patient selection, the healthcare system of the respective country, and the surgical technique were identified as key influencing factors [40].

## RESULTS

A total of 15 primary studies on PFA were included in this meta‐analysis, comprising 1.477 implanted PFAs of 1248 participants. The studies incorporated 967 (77.5%) female patients and 281 (22.5%) male patients [1, 2, 6, 9, 13, 17, 19, 22, 26, 27, 28, 32, 39, 41, 47]. Fourteen studies were originally published in English, while one study was available in both Spanish (original) and English. For analytic standardization, each study contributed the survival time point closest to its median follow‐up (Figure 1 and Table 1).

**Figure 1:**
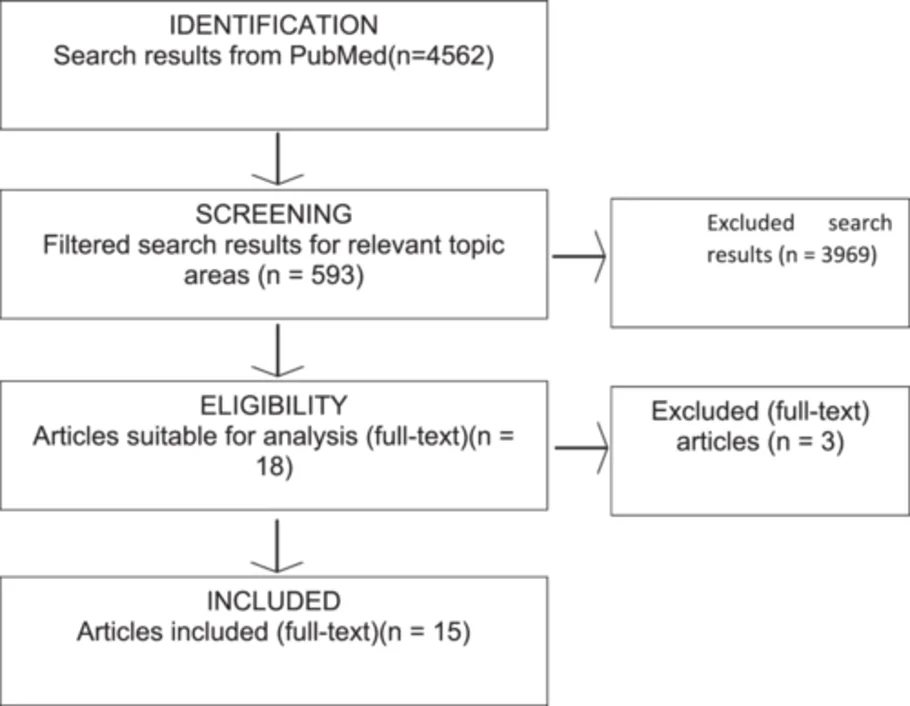
The flowchart illustrates the search results based on the search string. After identifying the relevant studies, they were filtered according to relevant topics. Subsequently, the studies were checked for their suitability for analysis, with all criteria needing to be met before a total of 15 studies could be included.

**Table 1:** The 15 included studies were analyzed based on the following points: Study and year of publication, implant type, implant design, number of PFAs, number of PFAs at final follow‐up, % male of cohort, survival % (time point), investigated parameters ROBINS‐I‐Tool, follow‐up category and study type.

Study and year of publication	Implant‐type	Implant‐design	Number of PFAs	Number of PFAs at final follow‐up	Final follow‐up	% Male of cohort	Average age	Survival % (time point)	Investigated parameters	ROBINS‐I‐Tool	Follow‐up category	Study type
Akhbari et al. 2015	Avon	Onlay‐Design	61	55	5 Years (1–10); 10 years	10.53 (6/57)	66.1 (NR)	96.2% (5 years); 88.9% (10 years)	Complications + Survival	Critical	Mid‐term	Independent study
Ahearn et al. 2016	Journey	Onlay‐Design	101	90	7 Years (5–9)	19.80 (20/101)	60 (26–86)	88% (7 years)	Complications + Survival	Critical	Mid‐term	Independent study
Bohu et al. 2019	Hermes	Onlay‐Design	74	70	8 Years (2–20)	18.75 (12/64)	60 (31–82)	85.7% (8 years)	Complications	Critical	Long‐term	Independent study
Clement et al. 2019	Avon	Onlay‐Design	54	54	10 Years (8–15)	9.26 (5/54)	62 (NR)	94.2% (5 years); 92.3% (10 years)	Complications + Survival	Critical	Long‐term	Independent study
deDeugd et al. 2017	Avon	Onlay‐Design	75	75	3 Years (2–10)	12.00 (9/75)	51 (36–81)	94.7% (5 years)	Complications	Critical	Mid‐term	Independent study
Hoogervorst et al. 2015	Richards' II	Inlay‐Design	33	28	6 Years (2–18)	20.83 (5/24)	47 (32–81)	73% (10 years)	Complications + Survival	Critical	Mid‐term	Independent study
Imhoff et al. 2019	Hemi‐CAP	Inlay‐Design	37	24	5 Years (2–5)	68.57 (24/35)	49 (22–79)	83% (5 years)	Complications	Serious	Mid‐term	Independent study
Konan and Haddad 2016	Avon	Onlay‐Design	51	48	7 Years (5–11)	61.7 (29/47)	57 (37–69)	96.1% (11 years)	Complications	Critical	Long‐term	Developer study
Metcalfe et al. 2018	Avon	Onlay‐Design	558	390	18 Years (2–18)	18.41 (79/429)	59 (25–92)	77.3% (10 years); 67.4% (15 years)	Complications + Survival	Serious	Long‐term	Developer study
Middleton et al. 2018	Avon	Onlay‐Design	103	103	6 Years (3–14)	34.95 (36/103)	60 (34–82)	89% (5 years); 86% (10 years)	Complications	Critical	Mid‐term	Independent study
Osarumwense et al. 2017	ZimmerBiomet	Onlay‐Design	52	49	3 Years (2–5)	25.00 (9/36)	59 (39–80)	95.6% (5 years)	Complications	Critical	Mid‐term	Independent study
Rammohan et al. 2019	Journey	Onlay‐Design	103	102	6 Years (2–11)	31.64 (25/79)	58 (42–78)	94.3% (7 years)	Complications + Survival	Critical	Mid‐term	Independent study
Romagnoli and Marullo 2017	ZimmerBiomet	Onlay‐Design	108	105	6 Years (3–8)	18.82 (16/85)	68 (39–88)	95.2% (6 years)	Complications	Critical	Long‐term	Independent study
Wang et al. 2023	YLQ	Onlay‐Design	46	28	24 Years (19–30)	13.16 (5/38)	55 (36–73)	83.6% (15 years); 76.8% (20 years); 59.4% (25 years)	Complications	Critical	Long‐term	Independent study
Martínez‑Sañudo et al. 2023	Avon and ZimmerBiomet	Onlay‐Design	21	21	Mean 9.7 years (1–14)	4.76 (1/21)	Median 63 (20–78)	73.5% (10 years)	Complications + Survival	Serious	Long‐term	Independent study

Abbreviation: PFA, patellofemoral arthroplasty.

The fixed‐effects model yielded a pooled revision rate of 1.95/100 CY (95% CI = 1.73–2.19), correlating to a 19.5% 10‐year revision rate. Using a DerSimonian–Laird random‐effects model, the pooled rate was 1.59/100 CY (95% CI = 1.24–2.04), which correlates with a 15.9% revision rate at 10 years.

In subgroup analyses, ‘developer’ studies demonstrated a pooled revision rate of 1.28 revisions per 100 CY (95% CI = 0.43–3.21), whereas independent studies showed a higher pooled rate of 1.75 revisions per 100 CY (95% CI = 1.45–2.10). Stratification by implant design revealed lower revision rates for onlay implants (13 out of 15 studies), with a pooled random‐effects estimate of 1.43 revisions per 100 CY (95% CI = 1.09–1.88), compared with inlay designs (2 out of 15 studies), which demonstrated a higher pooled rate of 2.97 revisions per 100 CY (95% CI = 1.80–4.91). When analyzed by follow‐up duration, the studies showed comparable revision rates between mid‐term studies (≤7 years; 8 studies), with a pooled rate of 1.52 revisions per 100 CY (95% CI = 1.18–1.98), and long‐term studies (>7 years; 7 studies), which yielded a pooled rate of 1.78 revisions per 100 CY (95% CI = 1.39–2.29) (Figure 2).

**Figure 2:**
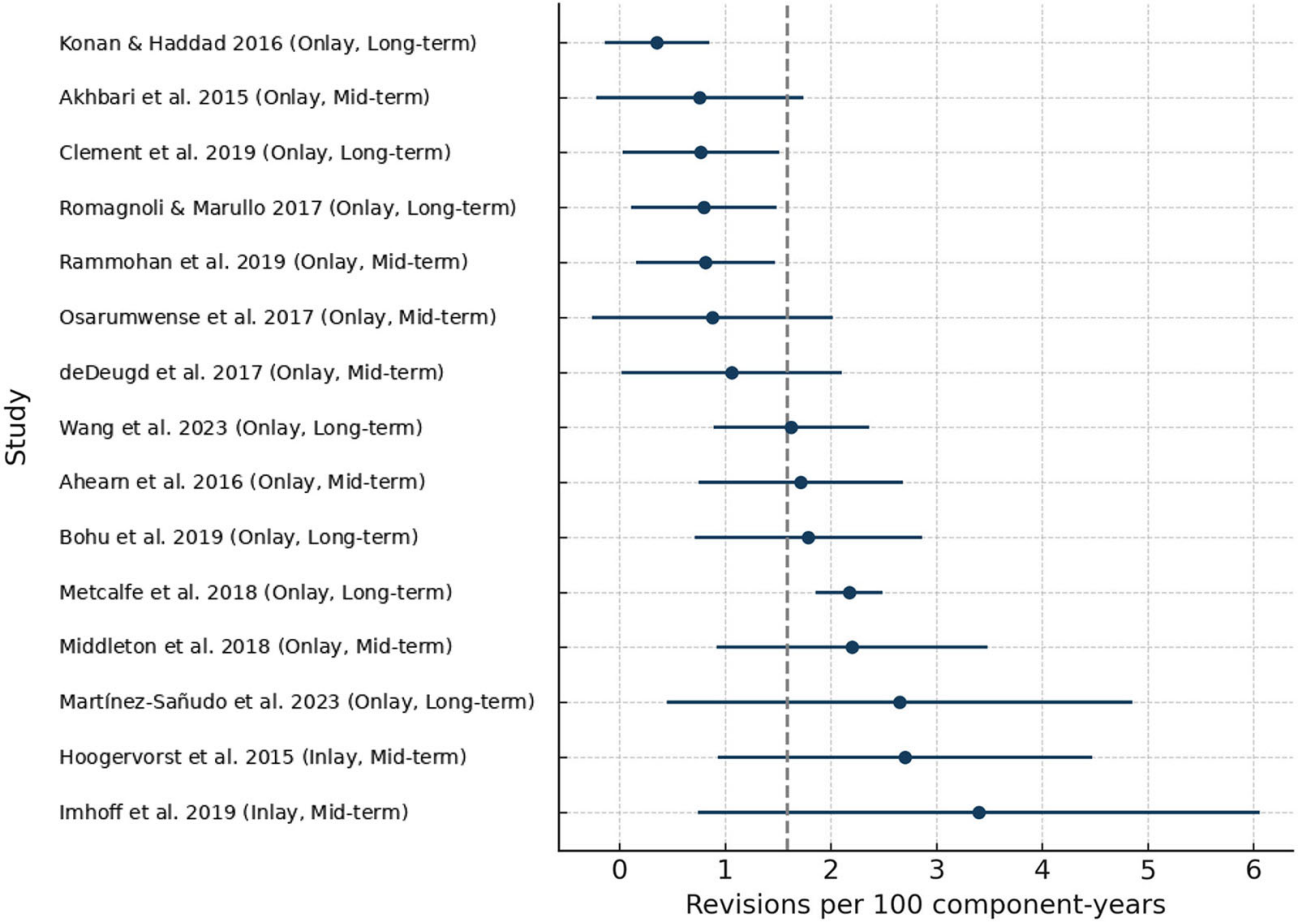
Forest plot of the 15 included PFA studies. Outcomes are expressed as revisions per 100 CY with 95% confidence intervals. The dashed vertical line denotes the random‐effects pooled estimate (1.59 per 100 CY). CY, component‐years; PFA, patellofemoral arthroplasty.

Moderate between‐study heterogeneity was observed (*Q* = 29.57; df = 14; *I* ^2^ = 52.7%; *τ* ^2^ = 0.0973). This variability is largely attributable to differences in implant design and the heterogeneity of follow‐up horizons across studies.

Heterogeneity was lower in the subgroup analysis among mid‐term studies (*Q* = 9.8; df = 7; *I* ^2^ = 28.6%; *τ* ^2^ = 0.031) and substantially higher among long‐term studies (*Q* = 19.2; df = 6; *I* ^2^ = 68.7%; *τ* ^2^ = 0.112) (Figure 3).

**Figure 3:**
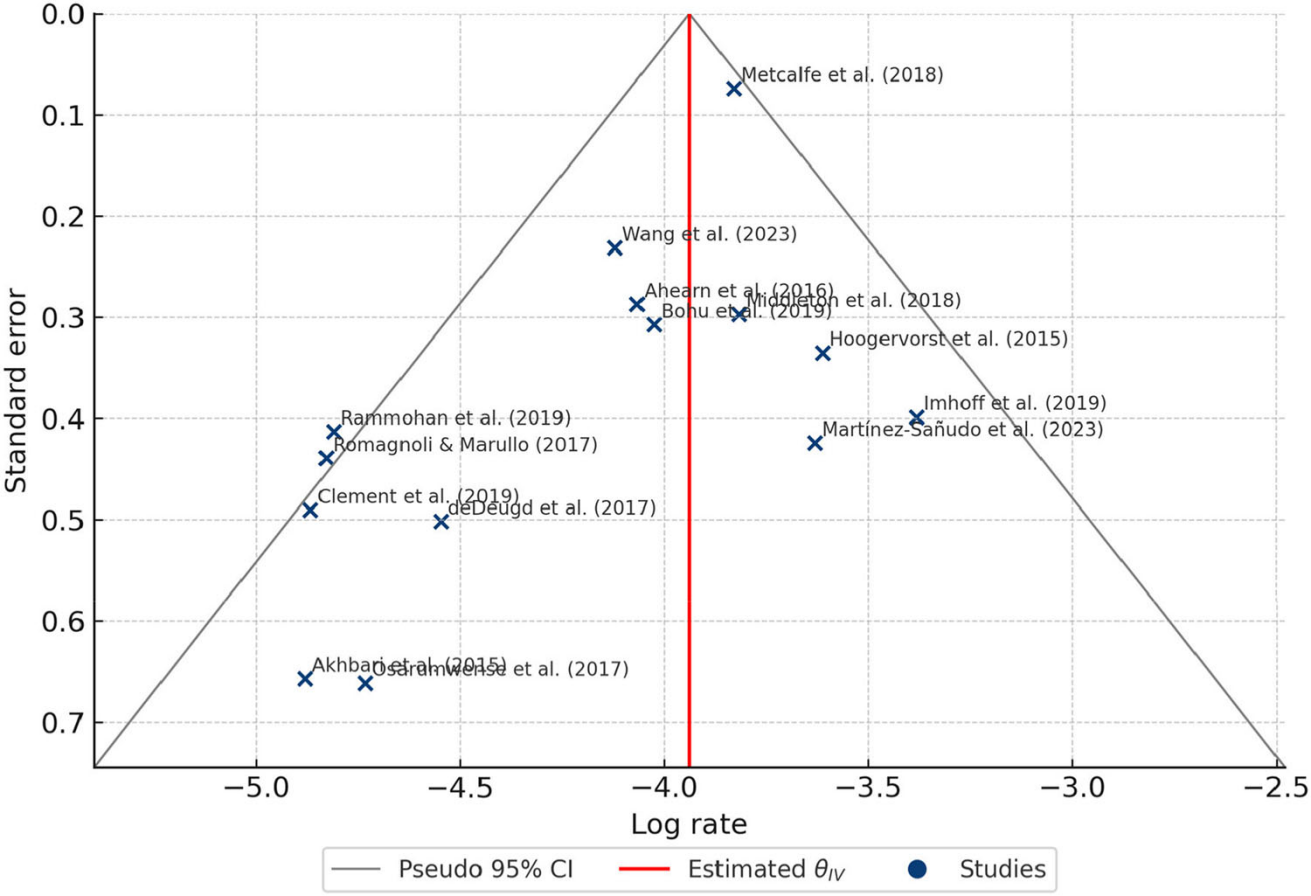
Funnel plot of small‐study effects. Each dot represents one of the 15 included studies, plotted as the log revision rate (*x*‐axis; events per CY against its standard error (*y*‐axis; inverted scale so precision increases upward). The red vertical line marks the inverse‐variance fixed‐effects estimate. Grey lines depict the pseudo 95% confidence limits. CY, component‐years.

Overall, the ROBINS‐I risk of bias across 15 non‐randomized PFA studies was relatively high (Figure 4).

**Figure 4:**
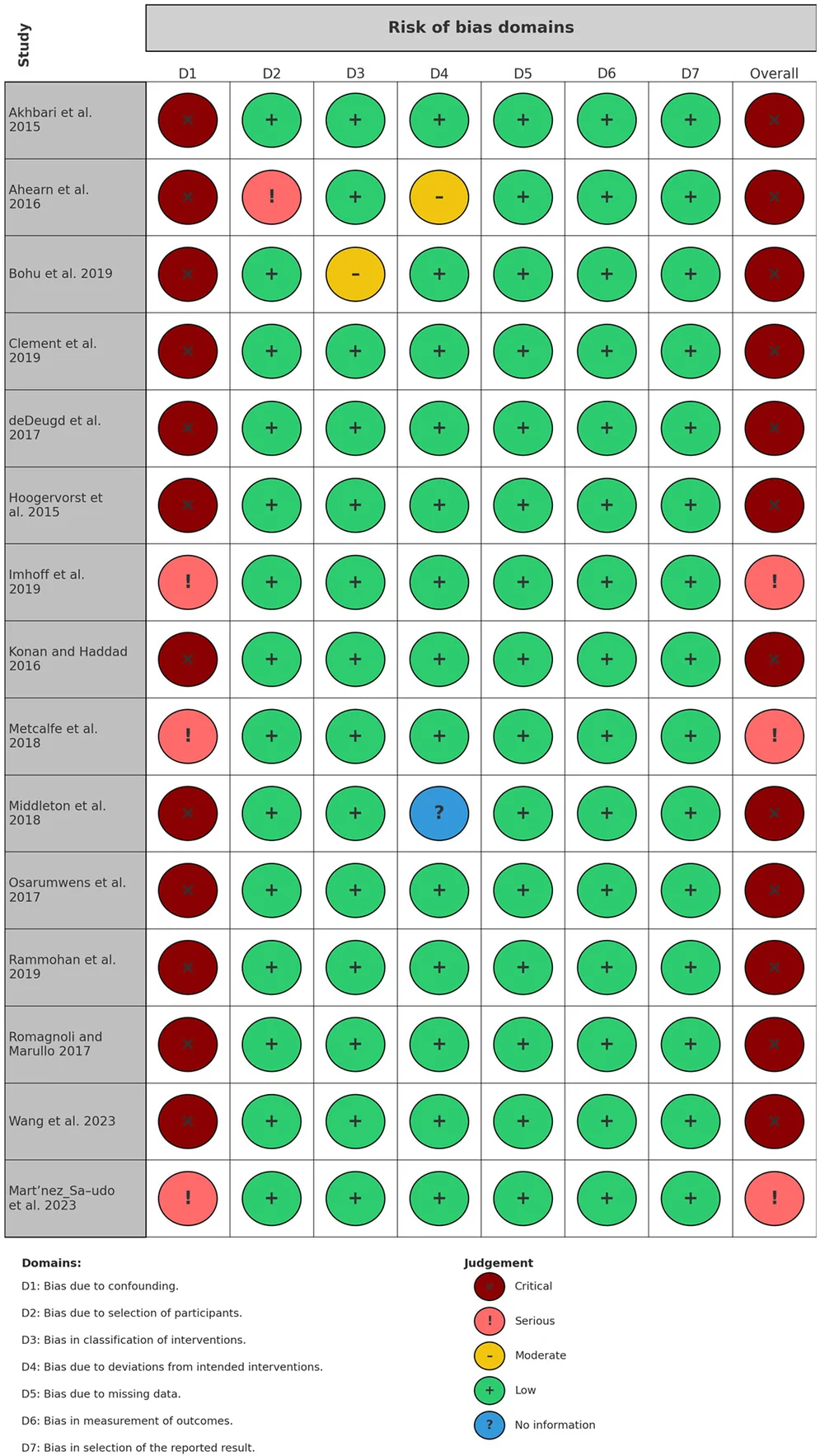
The percentage distribution of the ‘low risk’, ‘moderate risk’ and ‘serious risk’ ratings among the 15 included studies by using the ROBINS‐I tool. The listed seven domains were analyzed individually for each study, and subsequently, the overall risk was determined.

Across national arthroplasty registries, the pooled revision rate after PFA was 1.85/100 CY (95% CI = 1.775–1.931), based on a total exposure of 117.524 CY and 2178 revision events, which corresponds to a 10‐year revision probability of 18.53% (95% CI = 17.753–19.309) and an inferred 10‐year survivorship of 81.47% (95% CI = 80.691–82.247) (Table 2).

**Table 2:** Summary of the three national registries included in this meta‐analysis. With the primary endpoint, revision for any reason per 100 component‐years (CY).

Registry	Country	Years covered	CY	Revisions/100 CY (95% CI)	10‐year revision % (95% CI)	10‐year survivorship % (95% CI)	Reporting level
NJR (National Joint Registry)	United Kingdom	2015–2025	84.400	1.69 (1.625–1.758)	16.90 (16.25–17.58)	83.10 (82.42–83.75)	EAR/EUPHORIC Type A.1.1.1.1
NZJR (New Zealand Joint Registry)	New Zealand	2015–2025	6.264	1.92 (1.59–2.29)	19.20 (15.90–22.90)	80.80 (77.10–84.10)	EAR/EUPHORIC Type A.1.1.1.1
AOANJRR (Australian Orthopaedic Association National Joint Replacement Registry)	Australia	2015–2025	26.860	2.350 (2.035–2.695)	23.50 (20.35–26.95)	76.50 (73.05–79.65)	EAR/EUPHORIC Type A.1.1.1.1

## DISCUSSION

The most important finding of this study is that contemporary data suggest a more favourable survivorship profile of PFA compared with earlier reports. Using the same endpoints and methodology as Reihs et al., the pooled random‐effects revision rate was 1.59 revisions per 100 CY in clinical studies and 1.85 revisions per 100 CY in registry data. Although no formal statistical comparison was performed, these values are numerically lower than those previously reported, indicating a potential temporal trend towards improved implant survival.

The primary outcome of this study, revisions per 100 CY, indicates a more favourable survivorship profile of PFA compared with the preceding decade, with a literature‐derived rate of 1.59/100 CY versus 2.22/100 CY reported by Reihs et al. and a pooled registry rate of 1.85/100 CY versus 1.89–2.70/100 CY in the prior decade. These findings are supported by contemporary reports, which attribute the observed gains chiefly to optimized implant designs and refinements in surgical techniques, while the progression of osteoarthritis remains the predominant indication for revision [18, 42, 46]. This prompts consideration in everyday clinical practice of whether PFA, when appropriately indicated, is superior to TKA, given the lower 10‐year revision risk of TKA [10, 24, 31]. However, DeFrance et al. [12] reported a 10% patient dissatisfaction rate in a systematic review, which precludes a definitive answer to this debate. Furthermore, it is crucial to consider that CY‐based comparisons provide an average estimate of revision rates and cannot account for time‐varying failure mechanisms or differences in follow‐up distributions across studies.

The overall revision rate across studies was highly variable despite moderate heterogeneity, ranging from 0.355/100 CY to 3.400/100 CY. Notably, the Avon implant yielded the most favourable outcome, with 96.1% survivorship at 11 years. Given that this study was independent without developer involvement, certain biases can be ruled out. Nevertheless, the survival curve is directly affected by surgical experience, hospital setting, and other factors, which attenuates the strength of the conclusions and indicates that any truly superior implant should be identified through future multicenter studies [40].

When comparing clinical studies to national registry data, the revision rates are 1.59/100 CY versus 1.85/100 CY, corresponding to 10‐year revision rates of 15.9% and 18.5%, respectively. It is striking that registry‐derived revision rates are higher, a finding that aligns with current literature [40, 46]. This is readily explained by the fact that national registries reflect the entire population, including older, multimorbid patients, anatomically complex PFAs and less stringent indications, factors that directly increase the likelihood of revision. Registries also capture the full reality of care, encompassing low surgeon volumes and learning curves, variability in surgical technique and heterogeneous rehabilitation pathways. By contrast, the analyzed studies largely represent high‐volume specialist centres with substantial expertise that employ contemporary PFA implants, which in turn lower the probability of revision [15, 38, 46]. Accordingly, comparisons between registry data and clinical studies should be interpreted with caution, and conclusions regarding PFA survivorship must remain descriptive rather than definitive.

Given the observed increase in PFA survival over the last decade, recent studies have examined the role of robotic assistance in enhancing surgical precision. Early evidence suggests that robot‐assisted techniques reduce variability in component placement compared with conventional methods, potentially minimizing maltracking, edge loading and early wear, which are crucial contributors to early failure. Improved accuracy may also preserve native bone and soft‐tissue balance, facilitating more predictable kinematics and reducing revision rates. While long‐term data are still emerging, the integration of robotics appears to be a promising adjunct that could contribute to the trend towards enhanced implant longevity observed in recent registries and cohort studies [21, 35, 48]. Beyond technical advancements, changes in indication practice may also have influenced contemporary outcomes. An increasing emphasis on confirmed isolated patellofemoral osteoarthritis, the absence of relevant tibiofemoral degeneration and appropriate limb alignment reflects a more restrictive selection strategy. Given that progression of tibiofemoral osteoarthritis remains a leading cause of revision, stricter adherence to these criteria may partially contribute to the observed numerical reduction in revision rates [3, 36, 42, 43].

According to the ROBINS‐I assessment, 12 of the included studies were rated as having a critical risk of bias and three as having a serious risk of bias. These ratings were predominantly driven by potential bias due to confounding, reflecting the non‐randomized design of the available evidence and limited control for relevant patient‐, implant‐ and surgeon‐related factors. While such studies remain highly informative for capturing real‐world PFA performance, particularly in the context of evolving implant designs and surgical techniques, the presence of confounding limits the precision with which causal inferences can be drawn from the pooled estimates. Consequently, the reported revision rates should be interpreted as descriptive summaries of observed revision rates rather than definitive effect estimates, and their clinical interpretation should be considered alongside registry data and future comparative studies with improved methodological control.

Several limitations must be considered when interpreting these findings. First, all included studies were non‐randomized cohort series and were predominantly rated at critical risk of bias according to ROBINS‐I. Confounding by indication, differences in implant selection, surgeon experience and institutional case volume may have influenced revision rates and cannot be fully adjusted for within the available data structure. Consequently, the pooled estimates should be interpreted as descriptive summaries of observed revision rates rather than causal effect estimates.

Second, the use of revisions per 100 CY assumes a constant hazard rate over time and does not account for time‐dependent failure mechanisms. Early technical failures and late progression of tibiofemoral osteoarthritis may follow different hazard patterns that are not fully captured by CY‐based aggregation. Furthermore, heterogeneous follow‐up durations across studies may influence the stability of pooled estimates despite random‐effects modelling.

Third, although potential cohort overlap was carefully assessed, complete exclusion of overlapping patient populations cannot be guaranteed due to incomplete reporting of recruitment periods and loss‐to‐follow‐up accounting in some series. Informative censoring and inconsistent reporting of deaths or secondary procedures may therefore have affected the calculated revision rates.

Fourth, no formal statistical comparison with the prior decade analysis was performed because individual patient‐level data were unavailable. The observed differences should therefore be interpreted as numerical trends rather than statistically confirmed improvements.

## CONCLUSION

This meta‐analysis demonstrates that modern PFA has achieved a more favourable survivorship profile over the past decade, particularly with onlay designs. Proper patient selection and registry‐based surveillance remain key to further optimization.

## AUTHOR CONTRIBUTIONS

**Clemens Clar**: Conceptualization; methodology; analysis of data; investigation; data curation; writing—original draft preparation. **Maximilian Budin**: Conceptualization; methodology; analysis of data; investigation; data curation; supervision; writing—review and editing. **Laura Rasic**: Conceptualization; methodology; analysis of data; investigation; data curation; supervision; writing—review and editing. **Andreas Leithner**: Conceptualization; methodology; analysis of data; investigation; data curation. **Julia von der Linden**: Conceptualization; methodology; analysis of data; investigation; data curation; supervision; writing—review and editing. **Patrick Sadoghi**: Conceptualization; methodology; analysis of data; investigation; data curation; supervision; writing—review and editing.

## CONFLICT OF INTEREST STATEMENT

Andreas Leithner and Patrick Sadoghi report institutional educational grants by Medacta, Implantcast and Johnson & Johnson without any relation to this work. The remaining authors declare no conflicts of interest.

## ETHICS STATEMENT

The authors have nothing to report.

## Data Availability

The data that support the findings of this study are not openly available due to reasons of sensitivity, but are available from the corresponding author upon reasonable request.

## References

[1] Ahearn N , Metcalfe AJ , Hassaballa MA , Porteous AJ , Robinson JR , Murray JR , et al. The journey patellofemoral joint arthroplasty: a minimum 5 year follow‐up study. Knee. 2016;23(5):900–904. 10.1016/j.knee.2016.03.004 27131405

[2] Akhbari P , Malak T , Dawson‐Bowling S , East D , Miles K , Butler‐Manuel PA . The Avon patellofemoral joint replacement: mid‐term prospective results from an independent centre. Clin Orthop Surg. 2015;7(2):171–176. 10.4055/cios.2015.7.2.171 PMC4515456 26217462

[3] Aweid O , Ahearn N , Metcalfe AJ , Eldridge J , Porteous A , Murray JR , et al. A radiological index that influences the outcome following patellofemoral joint arthroplasty: the anterior trochlea offset ratio. Knee Surg Sports Traumatol Arthrosc. 2023;31(4):1412–1419. 10.1007/s00167-022-07085-1 PMC10049942 36098748

[4] Bendixen NB , Eskelund PW , Odgaard A . Failure modes of patellofemoral arthroplasty‐registries vs. clinical studies: a systematic review. Acta Orthop. 2019;90(5):473–478. 10.1080/17453674.2019.1634865 PMC6746256 31259645

[5] Blazina ME , Fox JM , PLZZO WD , Broukhim B , Ivey FM . Patellofemoral replacement. Clin Orthop Relat Res. 1979;144:98–102. 535258

[6] Bohu Y , Klouche S , Sezer HB , Gerometta A , Lefevre N , Herman S . Hermes patellofemoral arthroplasty: annual revision rate and clinical results after two to 20 years of follow‐up. Knee. 2019;26(2):484–491. 10.1016/j.knee.2019.01.014 30797677

[7] Bond EC , Stauffer TP , Hendren S , Amendola A . Modern patellofemoral arthroplasty. JBJS Rev. 2023;11(9):e23. 10.2106/JBJS.RVW.23.00071 37656827

[8] Clar C , Leitner L , Koutp A , Hauer G , Rasic L , Leithner A , et al. The worldwide survival rate of total hip arthroplasties is improving: a systematic comparative analysis using worldwide hip arthroplasty registers. EFORT Open Rev. 2024;9(8):745–750. 10.1530/EOR-23-0080 PMC11370712 39087499

[9] Clement ND , Howard TA , Immelman RJ , MacDonald D , Patton JT , Lawson GM , et al. Patellofemoral arthroplasty versus total knee arthroplasty for patients with patellofemoral osteoarthritis: equal function and satisfaction but higher revision rate for partial arthroplasty at a minimum eight years' follow‐up. Bone Joint J. 2019;101–B(1):41–46. 10.1302/0301-620X.101B1.BJJ-2018-0654.R2 30601045

[10] Clement ND , Yapp LZ , Scott CEH . The estimated lifetime risk of revision after primary knee arthroplasty is influenced by implant design and patient age: data from the National Joint Registries. J Arthroplasty. 2025;40(6):1547–1553.e5. 10.1016/j.arth.2024.11.054 39631511

[11] Davies AP , Vince AS , Shepstone L , Donell ST , Glasgow MM . The radiologic prevalence of patellofemoral osteoarthritis. Clin Orthop Relat Res. 2002;402(402):206–212. 10.1097/00003086-200209000-00020 12218486

[12] DeFrance MJ , Scuderi GR . Are 20% of patients actually dissatisfied following total knee arthroplasty? A systematic review of the literature. J Arthroplasty. 2023;38(3):594–599. 10.1016/j.arth.2022.10.011 36252743

[13] deDeugd CM , Pareek A , Krych AJ , Cummings NM , Dahm DL . Outcomes of patellofemoral arthroplasty based on radiographic severity. J Arthroplasty. 2017;32(4):1137–1142. 10.1016/j.arth.2016.11.006 27979409

[14] EFORT Website for European Arthroplasty Registers , EARWelcome. 2025. http://www.ear.efort.org. Accessed 3 Oct 2025.

[15] Ennis HE , Phillips JLH , Jennings JM , Dennis DA . Patellofemoral arthroplasty. J Am Acad Orthop Surg. 2023;31(19):1009–1017. 10.5435/JAAOS-D-23-00022 37364255

[16] Havelin LI , Vollset SE , Engesæter LB . Revision for aseptic loosening of uncemented cups in 4,352 primary total hip prostheses: a report from the Norwegian Arthroplasty Register. Acta Orthop Scand. 1995;66(6):494–500. 10.3109/17453679509002301 8553814

[17] Hoogervorst P , de Jong RJ , Hannink G , van Kampen A . A 21% conversion rate to total knee arthroplasty of a first‐generation patellofemoral prosthesis at a mean follow‐up of 9.7 years. Int Orthop. 2015;39(9):1857–1864. 10.1007/s00264-015-2941-1 26231494

[18] Hoogervorst P , Arendt EA . Patellofemoral arthroplasty: expert opinion. J Exp Orthop. 2022;9(1):24. 10.1186/s40634-022-00457-z PMC8897539 35244809

[19] Imhoff AB , Feucht MJ , Bartsch E , Cotic M , Pogorzelski J . High patient satisfaction with significant improvement in knee function and pain relief after mid‐term follow‐up in patients with isolated patellofemoral inlay arthroplasty. Knee Surg Sports Traumatol Arthrosc. 2019;27(7):2251–2258. 10.1007/s00167-018-5173-2 30276437

[20] Jagadeesh N , Sales‐Fernández R , Pammi S , Kariya A . Functional outcomes, survival rate, and complications of patellofemoral arthroplasty: mid‐term results from independent center. Cureus. 2022;14(11):e31945. 10.7759/cureus.31945 PMC9794910 36582575

[21] Katzman JL , Buehring W , Haider MA , Connolly P , Schwarzkopf R , Fernandez‐Madrid I . Clinical outcomes of patellofemoral arthroplasty: robotic assistance produces superior short and mid‐term outcomes. Arch Orthop Trauma Surg. 2024;144(9):4017–4028. 10.1007/s00402-024-05263-z 38492064

[22] Konan S , Haddad FS . Midterm outcome of Avon patellofemoral arthroplasty for posttraumatic unicompartmental osteoarthritis. J Arthroplasty. 2016;31(12):2657–2659. 10.1016/j.arth.2016.06.005 27522543

[23] Labek G , Neumann D , Agreiter M , Schuh R , Böhler N . Impact of implant developers on published outcome and reproducibility of cohort‐based clinical studies in arthroplasty. J Bone Jt Surg. 2011;93(Suppl 3):55–61. 10.2106/JBJS.K.01108 22262425

[24] Leitner L , Hauer G , Rasic L , Clar C , Leithner A , Sadoghi P . Development of application and surgical technique of total knee arthroplasties: a systematic comparative analysis using worldwide registers. Arch Orthop Trauma Surg. 2024;144(2):855–859. 10.1007/s00402-023-05089-1 PMC10822784 37843609

[25] Lewis PL , Tudor F , Lorimer M , McKie J , Bohm E , Robertsson O , et al. Short‐term revision risk of patellofemoral arthroplasty is high: an analysis from eight large arthroplasty registries. Clin Orthop Relat Res. 2020;478(6):1222–1231. 10.1097/CORR.0000000000001268 PMC7319370 32348089

[26] Martínez‐Sañudo B , Fornell S , Vallejo M , Domecq G . Midterm outcomes of patellofemoral arthroplasty. Rev Esp Cir Ortop Traumatol. 2023;67(4):317. English, Spanish. 10.1016/j.recot.2022.12.016 36574834

[27] Metcalfe AJ , Ahearn N , Hassaballa MA , Parsons N , Ackroyd CE , Murray JR , et al. The Avon patellofemoral joint arthroplasty: two‐ to 18‐year results of a large single‐centre cohort. Bone Joint J. 2018;100–B(9):1162–1167. 10.1302/0301-620X.100B9.BJJ-2018-0174.R1 30168762

[28] Middleton SWF , Toms AD , Schranz PJ , Mandalia VI . Mid‐term survivorship and clinical outcomes of the Avon patellofemoral joint replacement. Knee. 2018;25(2):323–328. 10.1016/j.knee.2018.01.007 29475782

[29] Moher D , Liberati A , Tetzlaff J , Altman DG . Preferred reporting items for systematic reviews and meta‐analyses: the PRISMA statement. PLoS Med. 2009;6(7):e1000097. 10.1371/journal.pmed.1000097 PMC2707599 19621072

[30] Odgaard A , Madsen F , Kristensen PW , Kappel A , Fabrin J . The Mark Coventry Award: patellofemoral arthroplasty results in better range of movement and early patient‐reported outcomes than TKA. Clin Orthop Relat Res. 2018;476(1):87–100. 10.1007/s11999.0000000000000017 PMC5919242 29529622

[31] Olson NR , Parks NL , Nagda SS , McAsey CJ , Fricka KB . To cement or not? Ten‐year results of a prospective, randomized study comparing cemented versus cementless total knee arthroplasty. J Arthroplasty. 2025;40(10):2630–2636. 10.1016/j.arth.2025.04.076 40339944

[32] Osarumwense D , Syed F , Nzeako O , Akilapa S , Zubair O , Waite J . Patellofemoral joint arthroplasty: early results and functional outcome of the Zimmer Gender Solutions Patello‐Femoral Joint System. Clin Orthop Surg. 2017;9(3):295–302. 10.4055/cios.2017.9.3.295 PMC5567024 28861196

[33] Pabinger C , Bridgens A , Berghold A , Wurzer P , Boehler N , Labek G . Quality of outcome data in total hip arthroplasty: comparison of registry data and worldwide non‐registry studies from 5 decades. HIP Int. 2015;25(5):394–401. 10.5301/hipint.5000239 25837781

[34] Pabinger C , Lumenta DB , Cupak D , Berghold A , Boehler N , Labek G . Quality of outcome data in knee arthroplasty. Acta Orthop. 2015;86(1):58–62. 10.3109/17453674.2014.961119 PMC4366675 25191934

[35] Pacchiarotti G , Todesca A , Coppola M , Gumina S . Robotic‐assisted patellofemoral arthroplasty provides excellent implant survivorship and high patient satisfaction at mid‐term follow‐up. Int Orthop. 2024;48(8):2055–2063. 10.1007/s00264-024-06224-2 PMC11246259 38819666

[36] Pagano A , Agostinone P , Alesi D , Caputo D , Neri MP , Grassi A , et al. Almost 79% survival rate at 10‐year follow‐up for the patellofemoral joint arthroplasty: an Italian prosthetic registry study. Knee Surg Sports Traumatol Arthrosc. 2024;32(6):1525–1530. 10.1002/ksa.12150 38529690

[37] Peng G , Liu M , Guan Z , Hou Y , Liu Q , Sun X , et al. Patellofemoral arthroplasty versus total knee arthroplasty for isolated patellofemoral osteoarthritis: a systematic review and meta‐analysis. J Orthop Surg Res. 2021;16(1):264. 10.1186/s13018-021-02414-5 PMC8048312 33858458

[38] Pisanu G , Rosso F , Bertolo C , Dettoni F , Blonna D , Bonasia D , et al. Patellofemoral arthroplasty: current concepts and review of the literature. Joints. 2017;5(4):237–245. 10.1055/s-0037-1606618 PMC5738475 29270562

[39] Rammohan R. R , Gupta S , Lee PYF , Chandratreya A . The midterm results of a cohort study of patellofemoral arthroplasty from a non‐designer centre using an asymmetric trochlear prosthesis. Knee. 2019;26(6):1348–1353. Erratum in: Knee. 2020;27(2):613. doi:10.1016/j.knee.2020.02.009. Rammohan R [corrected to Rammohan, R]. 10.1016/j.knee.2019.10.026 31791722

[40] Reihs B , Reihs F , Labek G , Hochegger M , Leithner A , Böhler N , et al. No bias for developer publications and no difference between first‐generation trochlear‐resurfacing versus trochlear‐cutting implants in 15,306 cases of patellofemoral joint arthroplasty. Knee Surg Sports Traumatol Arthrosc. 2018;26(9):2809–2816. 10.1007/s00167-017-4692-6 28840268

[41] Romagnoli S , Marullo M . Mid‐term clinical, functional, and radiographic outcomes of 105 gender‐specific patellofemoral arthroplasties, with or without the association of medial unicompartmental knee arthroplasty. J Arthroplasty. 2018;33(3):688–695. 10.1016/j.arth.2017.10.019 29129614

[42] Ryan SP , Stambough JB , Huddleston JI , Levine BR . Highlights of the 2023 American Joint Replacement Registry Annual Report. Arthroplast Today. 2024;26:101325. 10.1016/j.artd.2024.101325 PMC11239969 39006856

[43] Sava MP , Neopoulos G , Leica A , Hirschmann MT . Patellofemoral arthroplasty with onlay prosthesis leads to higher rates of osteoarthritis progression than inlay design implants: a systematic review. Knee Surg Sports Traumatol Arthrosc. 2023;31(9):3927–3940. 10.1007/s00167-023-07404-0 PMC10435614 37005940

[44] Sterne JA , Hernán MA , Reeves BC , Savović J , Berkman ND , Viswanathan M , et al. ROBINS‐I: a tool for assessing risk of bias in non‐randomised studies of interventions. BMJ. 2016;355:i4919. 10.1136/bmj.i4919 PMC5062054 27733354

[45] van der List JP , Chawla H , Zuiderbaan HA , Pearle AD . Survivorship and functional outcomes of patellofemoral arthroplasty: a systematic review. Knee Surg Sports Traumatol Arthrosc. 2017;25(8):2622–2631. 10.1007/s00167-015-3878-z 26590562

[46] Vella‐Baldacchino M , Chughtai D , Kow J , Carr C , Coyle A , Farrow‐Foster A , et al. Outcomes of patellofemoral joint arthroplasty: a systematic review of revision timelines and complication rates. J Orthop Surg Res. 2025;20(1):289. 10.1186/s13018-025-05592-8 PMC11917147 40098204

[47] Wang Y , Bian Y , Qian W . Long‐Term clinical results of patellofemoral arthroplasty for isolated patellofemoral osteoarthritis. J Orthop Surg. 2023;31(1):10225536231162832. 10.1177/10225536231162832 36888931

[48] Williamson TR , Robins S , Super JT , Oussedik S , Clement N . Robotic arm‐assisted patellofemoral joint arthroplasty achieves good short‐term survival and functional outcomes: a systematic review and meta‐analysis. Knee Surg Sports Traumatol Arthrosc. 2026;34:3646–3654. 10.1002/ksa.70301 41614388

